# Total Electron Detachment and Induced Cationic Fragmentation Cross Sections for Superoxide Anion (O_2_^−^) Collisions with Benzene (C_6_H_6_) Molecules

**DOI:** 10.3390/ijms23031266

**Published:** 2022-01-23

**Authors:** Carlos Guerra, Sarvesh Kumar, Fernando Aguilar-Galindo, Sergio Díaz-Tendero, Ana I. Lozano, Mónica Mendes, Juan C. Oller, Paulo Limão-Vieira, Gustavo García

**Affiliations:** 1 Instituto de Física Fundamental, Consejo Superior de Investigaciones Científicas, Serrano 113-bis, 28006 Madrid, Spain; carlosguerra@iff.csic.es; 2 Laboratório de Colisões Atómicas e Moleculares, CEFITEC, Departamento de Física, Faculdade de Ciências e Tecnologia, Universidade NOVA de Lisboa, 2829-516 Caparica, Portugal; s.kumar@campus.fct.unl.pt (S.K.); anita_ilm@iff.csic.es (A.I.L.); mf.mendes@fct.unl.pt (M.M.); plimaovieira@fct.unl.pt (P.L.-V.); 3 Donostia International Physics Center (DIPC), Paseo Manuel de Lardizabal 4, 20018 Donostia-San Sebastián, Spain; fernando.aguilar@dipc.org; 4 Departamento de Química, Módulo 13, Universidad Autónoma de Madrid, 28049 Madrid, Spain; 5 Condensed Matter Physics Center (IFIMAC), Universidad Autónoma de Madrid, 28049 Madrid, Spain; 6 Institute for Advanced Research in Chemical Science (IAdChem), Universidad Autónoma de Madrid, 28049 Madrid, Spain; 7 Centro de Investigaciones Energéticas Medioambientales y Tecnológicas (CIEMAT), Avenida Complutense 22, 28040 Madrid, Spain; jc.oller@ciemat.es; 8 Centre for Medical Radiation Physics, University of Wollongong, Wollomgong, NSW 2522, Australia

**Keywords:** anion–molecule collisions, electron detachment cross sections, positive ion-induced fragmentation, molecular dynamics

## Abstract

In this study, novel experimental total electron detachment cross sections for O_2_^−^ collisions with benzene molecules are reported for the impact energy range (10–1000 eV), as measured with a transmission beam apparatus. By analysing the positively charged species produced during the collision events, relative total ionisation cross sections were derived in the incident energy range of 160–900 eV. Relative partial ionisation cross sections for fragments with *m*/*z* ≤ 78 u were also given in this energy range. We also confirmed that heavier compounds (*m*/*z* > 78 u) formed for impact energies between 550 and 800 eV. In order to further our knowledge about the collision dynamics governing the fragmentation of such heavier molecular compounds, we performed molecular dynamics calculations within the framework of the Density Functional Theory (DFT). These results demonstrated that the fragmentation of these heavier compounds strongly supports the experimental evidence of *m*/*z* = 39–42, 50, 60 (u) cations formation, which contributed to the broad local maximum in the total ionisation observed from 550 to 800 eV. This work reveals the reactivity induced by molecular anions colliding with hydrocarbons at high energies, processes that can take place in the interstellar medium under various local conditions.

## 1. Introduction

Benzene is one of the simplest and more stable aromatic ring molecules. It has been considered a prototype for the study of chemical reactions involving biomolecules, such as pyridine [1] and carbamates [2], a building block for the synthesis of carbon nano-cages [3] and a precursor of hydrocarbon structures (e.g., phenol, toluene, aniline, anthracene) with important technological applications [4]. The functionalization of fullerene-based nanostructures using the Density Functional Theory (DFT) has opened new investigations on their transport properties in biological media and their capability to be used as molecular sensors or drug carriers [5,6,7]. Additionally, oxygen superoxide anion, O_2_^−^, is one of the reactive oxygen species (ROS) which are responsible for numerous biochemical processes leading to oxidative damage in living organisms [8] as well as in materials [9]. In particular, 8-oxoguanine is frequently formed by the interaction of ROS with the guanine base in DNA under conditions of oxidative stress, yielding an efficient way of damaging DNA [10].

Due to benzene’s relevance for some of the applications listed above where charge transfer (electron transfer) is a pivotal mechanism in such reactions, electron scattering cross section data have been the subject of numerous theoretical and experimental studies. We recently reported an experimental and theoretical analysis of the total electron scattering cross section (TCS) with the aim of providing some reference data for modelling procedures connected with these applications [11]. Apart from TCS measurements and calculations, elastic scattering, vibrational excitation and electron attachment cross sections have also been published (see Ref. [11] and references therein). However, surprisingly, there is a lack of data on electron impact ionization and electronic excitation cross sections. Bull et al. in 2014 [12] evidenced this scarcity of data for aromatic molecules and provided new experimental data, which showed important discrepancies with the old measurements [13]. More recently, the double ionization of benzene by electron impact has been studied by Wolf et al. [14] and Sigaud and Montenegro [15], both presenting the remarkable probability of double ionization of benzene for increasing impact energies above 20 eV. Yet, being the projectile a superoxide anion, O_2_^−^, collision-induced ionization of a target molecule is certainly more complex to describe from the dynamical point of view of the interaction. To our knowledge, except for our recent study on the anomalous oxidation of benzene in collisions with superoxide anions [16], no experimental or theoretical data for O_2_^−^ collision with benzene are available in the literature. Notwithstanding, we report on absolute total electron detachment cross sections for such collision processes in the impact energy range of 200–900 eV. Measurements were obtained with a beam attenuation technique which has been previously described [16,17]. The total uncertainty limits of these results are within 6 and 10%. Additional experimental information on the induced cationic fragmentation, i.e., positively charged fragments with a mass-to-charge ratio (*m*/*z*) less than 78 u, is also given in terms of relative cross sections normalized to that of the parent ion formation with uncertainty limits within 10 and 20%. These relative fragmentation probabilities were also obtained by means of molecular dynamics calculations carried out in the framework of the density functional (DFT) theory. The remainder of this paper has been divided into the following sections: in Section 2, we present our experimental and theoretical data together with details of their associated uncertainties. These results are discussed and compared in Section 3. A detailed description of the theoretical and experimental methods used in this study are presented in Section 4. Finally, some conclusions are drawn in Section 5.

## 2. Results

The theoretical and experimental results of this study are presented in this section and discussed in Section 3, and a full description of the Material and Methods utilized to obtain these results is provided in Section 4.

### 2.1. Total Electron Detachment Cross Sections

**Figure 1:**
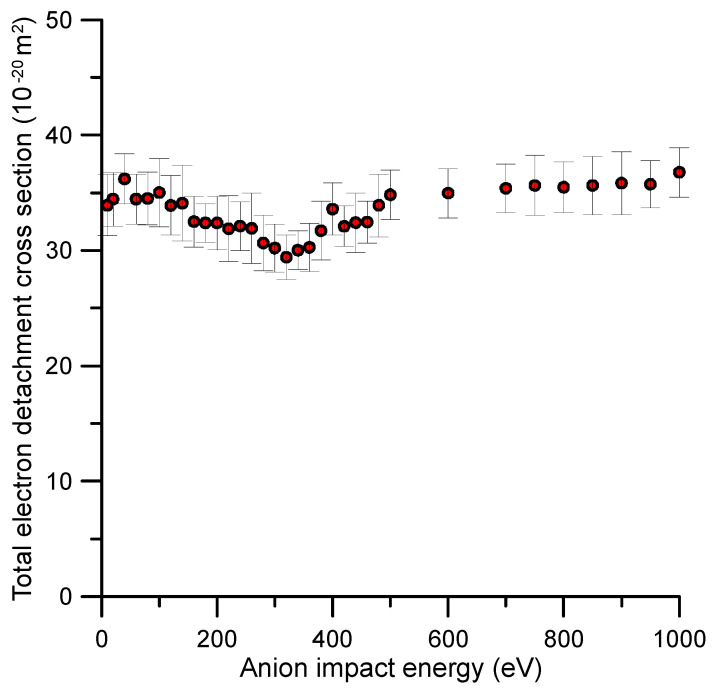
Total electron detachment cross section (TEDCS) for O_2_^−^ collisions with benzene (C_6_H_6_) as measured with the described transmission beam experimental setup (see Section 4 for details).

The experimental absolute total electron detachment cross sections (TEDCS) are presented in Table 1. Total uncertainty limits, according to the error estimation procedure described in Section 4, are also included in this table. These results are also plotted in Figure 1 together with their corresponding error bars.

**Figure 2:**
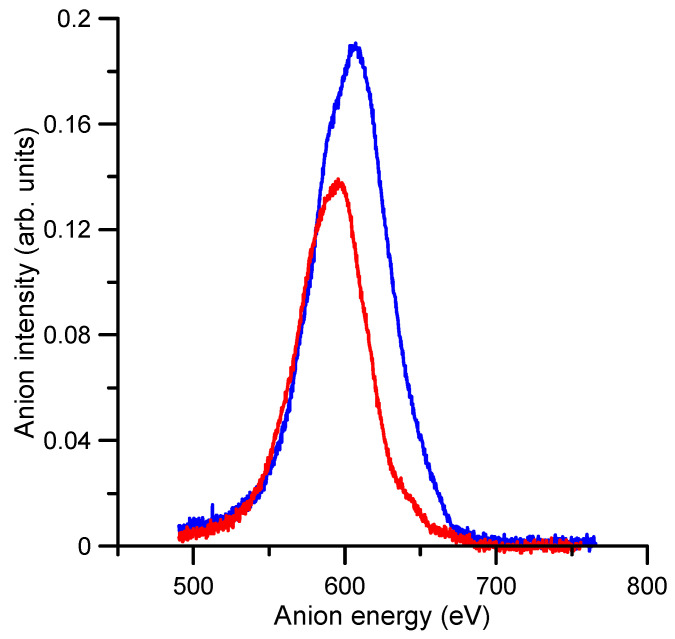
Intensity and energy of the transmitted anion beam with: 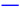, no benzene gas (0 mTorr) in the gas cell; 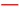, 1.2 mTorr of benzene in the gas cell.

As to the authors’ knowledge, no previous experimental or theoretical TEDCS data have been reported in the literature. If we compare these results with our recent measurements for O_2_^−^ collisions with N_2_ [17], we see that the average magnitude of the TEDCS in the common energy range (50–1000 eV) is in concordance with their respective molecular masses, i.e., 3–4 times higher for benzene than for nitrogen. In addition, the energy dependence of the TEDCSs shows some similarities for both molecular targets. In both cases, the magnitude of the cross section has some local maxima/minima superimposed to a relatively flat tendency. For N_2_, a prominent local maximum is shown around 200 eV (see Ref. [17]), while for benzene, two local maxima at 40 and 400 eV and a minimum around 320 eV are discernible. In contrast with nitrogen, these features are not well defined for benzene, and their respective magnitudes follow the flat tendency of the TEDCS if we consider their total uncertainty limits. These features should be connected with the excitation of specific energy levels of the target molecule. Figure 2 shows the time-of-flight spectra once calibrated in kinetic energy of the incident anion beam with and without benzene in the scattering region. As derived from this figure, the average energy loss by the primary beam after the collision with 1.2 mTorr of benzene was about 16 eV. This energy transferred was enough to induce single and double electronic excitations and/or ionisations to the benzene target molecule.

### 2.2. Induced Fragmentation of Benzene by O_2_^−^ Impact

**Figure 3:**
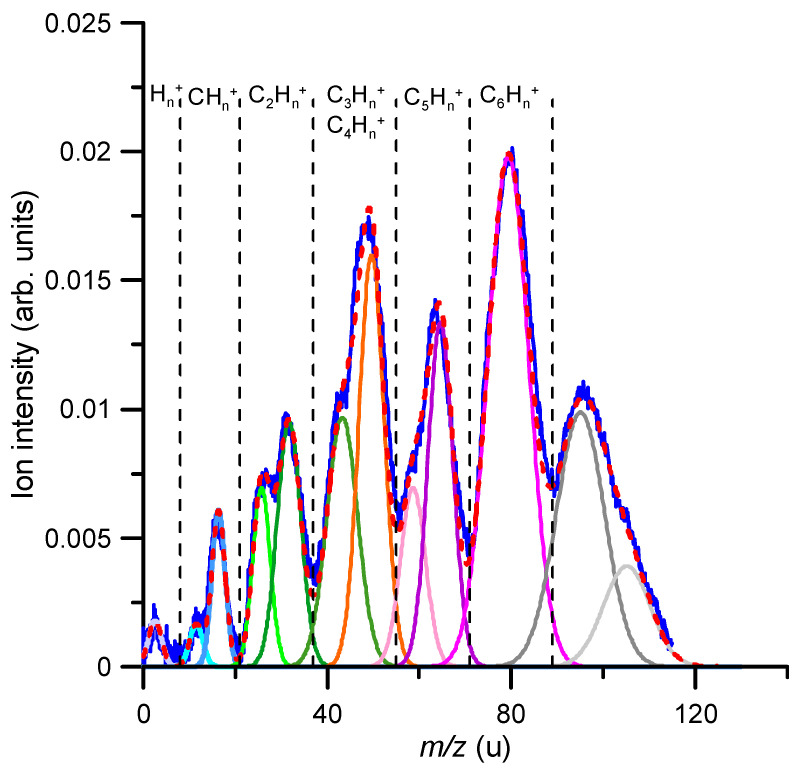
TOF mass spectrum of the positive ion-induced fragmentation of benzene in collisions with 850 eV oxygen anions (O_2_^−^). Gaussian fitting analysis: *m*/*z*(*u*) = 2 (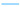); 12 (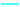); 16 (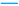); 26 (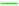); 32 (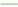); 42 (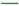); 50 (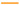); 60 (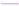); 65 (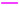); 78 (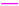); 94 (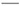); 110 (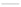); fit sum (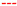).

The relative fragmentation yields of benzene cations formed after O_2_^−^ collisions in the energy range 160–900 eV were obtained by measuring the intensity of the TOF features, corresponding to identified fragments with *m*/*z* < 78 u, with respect to that of the parent ion (*m*/*z* = 78 u). A typical mass spectrum for an 850 eV impact energy is shown in Figure 3.

As described in Section 4, due to the specific anion formation process in the hollow cathode discharge, the time (mass) resolution was not enough to fully resolve each single fragment ion produced, but allowed distinguishing different fragment series clearly characterised by the number of carbon atoms constituting each group of cations. Nonetheless, using a gaussian fitting analysis procedure, we could identify the mass of the fragments that mainly contributed to each fragment group. For the anion impact energy shown in Figure 3 (850 eV), the most intense feature corresponds to the parent ion (C_6_H_6_^+^), and other significative ion fragment peaks were identified as C_4_H_2_^+^, C_5_H_5_^+^, C_2_H_6_^+^, C_3_H_6_^+^, C_2_H_2_^+^, C_5_^+^, CH_4_^+^, C^+^ and H/H_2_^+^ (in decreasing order of their relative intensities). Note that some features corresponding to ions heavier than the parent ion (*m*/*z* > 78 u) also appear in the mass spectrum. The formation of these heavy complexes can only be explained by the aggregation of oxygen molecules to some of the generated fragments, in particular [C_6_H_6_ + O] (m = 94 u) and [C_6_H_6_ + O_2_] (m = 110 u). The mechanisms by which these abducts are generated were investigated in a recent study, and the experimental evidence together with its theoretical justification can be found in Ref. [16]. We focused the present study on the formation of cationic fragments that were lighter than the parent ion, i.e., *m*/*z* < 78 u.

**Figure 4:**
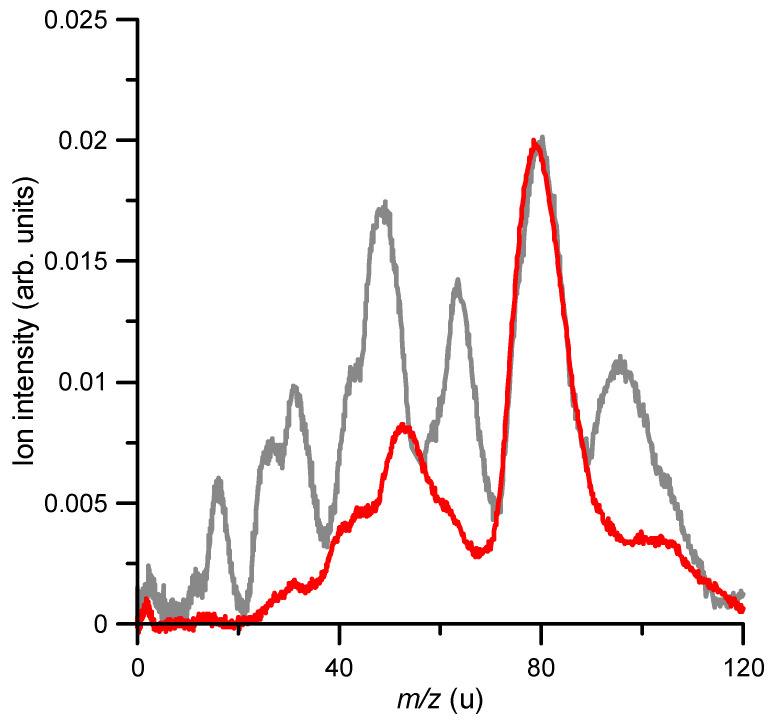
Positive ion-induced fragmentation by the superoxide anion beam (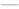) and the electron beam (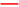), respectively, both at 850 eV impact energy.

It is interesting to compare the fragmentation induced by the anion beam to that produced by an electron beam in similar collision conditions. As described in Section 4, the experimental arrangement incorporated an electron gun normal to the anion beam, just in front of the TOF spectrometer used to analyse ions species of the generated fragments. Figure 4 shows the cationic fragmentation spectrum induced by 850 eV anion collisions (same as that shown in Figure 3) together with the induced fragmentation mass spectrum generated by an 850 eV electron beam. Both spectra are normalised to the parent ion (*m*/*z* = 78) intensity.

A close inspection of Figure 4 shows that superoxide anion projectiles produced a higher fragmentation pattern relative to electrons as projectiles. At 850 eV, 60% of the electron impact ionization corresponded to the formation of the parent ion, while in the case of O_2_^−^, it represented only 32.6% of the total ion yield. Thus, a clear enhancement of the target’s fragmentation induced by the anion beam was noted. In general, the relative intensity of the observed peaks obtained with the superoxide anion beam increased with respect to those obtained with the electron beam, but in particular, those with *m*/*z* = 65, 50 and 42 were especially enhanced, showing also a double-peak structure. This seemed to indicate that losses of CH, C_2_H_4_ and C_3_ radicals from the benzene ring were favoured in anion collisions.

**Figure 5:**
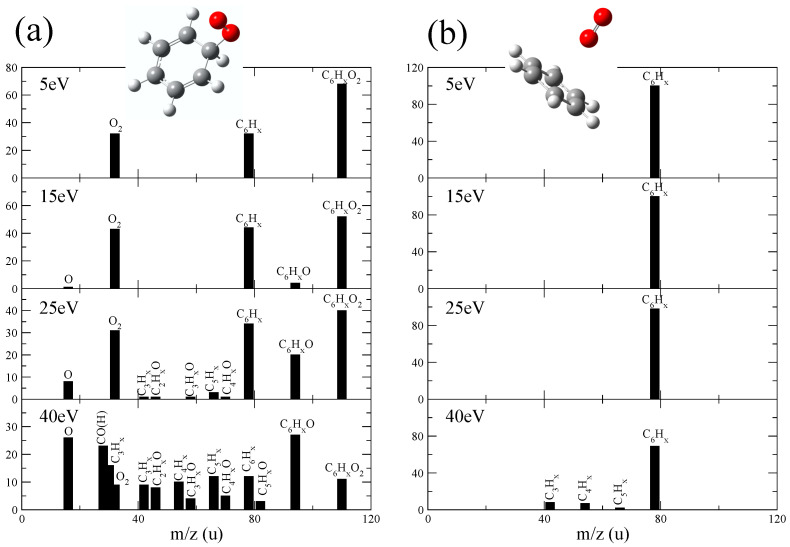
Simulated mass spectra for (**a**) [C_6_H_6_–O_2_]^+^ and (**b**) [C_6_H_6_ ··· O_2_]^+^ at different excitation energies derived from the molecular dynamics simulations.

In order to further our knowledge on the collision dynamics governing such cation formation, molecular dynamics calculations using the Density Functional Theory (DFT) were performed (see Section 4 for further details). In our previous work [16], we proposed a collision model with the formation of a stable complex in which O_2_ molecules remained bonded to the ionized benzene, [C_6_H_6_–O_2_]^+^. Non-associative collisions, where the O_2_ molecule did not remain bonded to benzene, might also occur. Therefore, we considered both situations and we ran two sets of trajectory calculations. The first one assumed chemical interaction and thus formation of a stable [C_6_H_6_–O_2_]^+^ molecule; therefore, these simulations started with the structure shown in Figure 5a. The starting point of the dynamics in the second one was a weakly bonded van der Waals complex [C_6_H_6_ ··· O_2_]^+^, shown in Figure 5b. Thus, both situations kept the same number of atoms but with a quite different bonding strength. Both initial structures were optimized and, thus, they were minima of the potential energy surfaces. The final results were obtained by computing statistics over the molecular dynamics simulations: 100 trajectories for each structure and each excitation energy value. Note that the excitation energy was randomly redistributed among the nuclear degrees of freedom in each trajectory. We considered that a fragment was created when the atoms forming this species were separated by at least 3 Å from other fragments in the last point of the trajectory.

In the simulated mass spectra in Figure 5, we included the percentage of trajectories where a given fragment appeared. In these spectra, we grouped all fragments with a different number of H atoms in a single peak, e.g., C_6_H_X_ included trajectories in which C_6_H_6_, C_6_H_5_, C_6_H_4_, etc. molecules were produced. The spectra corresponding to the weakly bonded [C_6_H_6_ ··· O_2_]^+^ were dominated by a single peak that corresponded to C_6_H_X_^+^ molecules. Further fragmentation was observed only for E_exc_ = 40 eV, in which a slightly amount of C_5_H_X_^+^, C_4_H_X_^+^ and C_3_H_X_^+^ could be noted. In the case of [C_6_H_6_–O_2_]^+^, a much richer spectrum was obtained, where oxygenated species were of particular importance: C_6_H_X_O_2_^+^ and C_6_H_X_O^+^, together with C_6_H_X_^+^, were the most prominent peaks at low excitation energies. At the highest excitation energy here considered, mono-oxygenated fragments, C_5_H_X_O^+^, C_4_H_X_O^+^, C_3_H_X_O^+^, gained in relative intensity. It is worth stressing that for [C_6_H_6_–O_2_]^+^, the relative intensity of non-oxygenated species, i.e., C_5_H_X_^+^, C_4_H_X_^+^, and C_3_H_X_^+^, was much higher than for the weakly bonded case.

**Figure 6:**
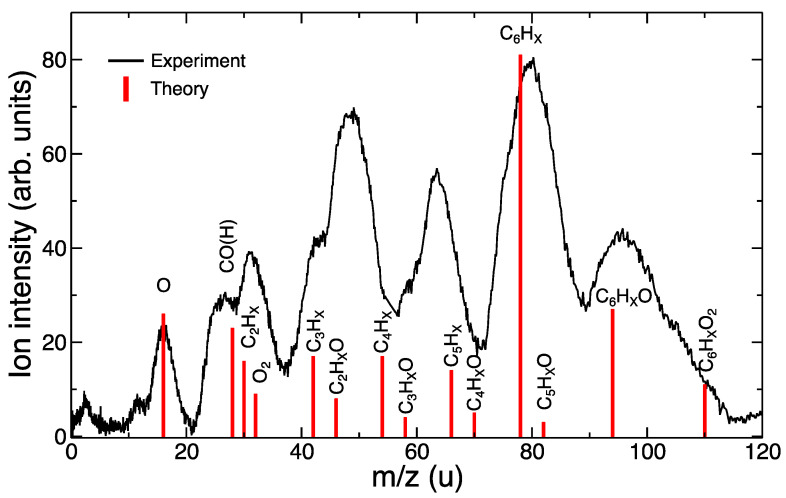
Comparison of experimental (collision energy = 850 eV) and theoretical (internal excitation energy = 40 eV) fragmentation spectra.

In the experimental mass spectrum, we could also discern double-peak structures, which we assigned to oxygenated and non-oxygenated species, e.g., the double-peak structure shown in the region 40–50 u, corresponded to C_3_H_X_ and C_2_H_X_O. Therefore, by only considering the fragmentation of [C_6_H_6_–O_2_]^+^, these double-peak structures can be explained. A comparison between the experimental measurements and the theoretical simulations is shown in Figure 6. For this purpose, we added both theoretical spectra obtained at 40 eV, i.e., we considered that both possibilities might occur, i.e., ionization and excitation of benzene (i) in a non-reactive collision and (ii) with formation of stable oxygenated species. A qualitative agreement was obtained, showing the validity of the model and corroborating the associative mechanism proposed in Ref. [16].

### 2.3. Relative Partial and Total Ionization Cross Sections of Benzene by O_2_^−^ Impact

**Figure 7:**
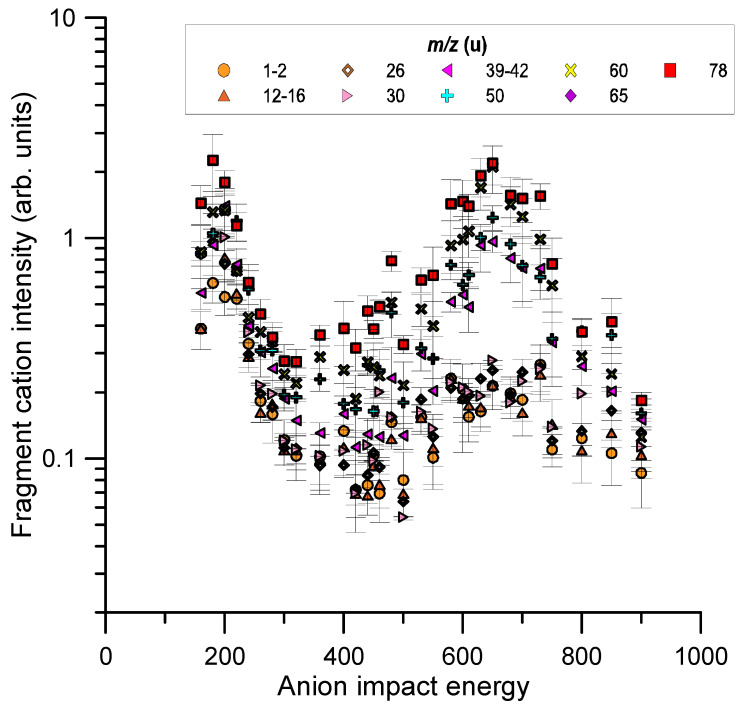
Relative cation intensities for *m*/*z* 78 u fragments induced by collisions with oxygen anions in the impact energy range (160–900 eV). See legend for the different cation species.

The relative intensities were normalised to the primary beam intensity and, according to Equation (2) (see Section 4, they accounted for the target gas pressure and geometry of the ion extraction region. However, as the ratio between the ion extracted beam and the actual primary beam intensity in the interaction region was not known, we could only provide relative values of the fragment yields. The energy dependence of these yields for the identified cation fragments is shown in Figure 7 for the whole impact energy range considered in this study (160–900 eV).

The fragment cation intensity distributions shown in Figure 3 were analysed as a function of the incident energy. For a given m/z fragment, its production cross section was given by the ratio between its detected ion intensity and the primary anion intensity divided by the target molecular density and the length of the ion extraction region (see Section 4 for technical details).

Following this procedure, we could in principle derive the absolute values of those partial ionisation cross sections. However, as already mentioned, we did not accurately know the ratio between the cation and the primary anion currents in the interaction region and therefore we could only report here on their relative cross section values. The error bars shown in Figure 7 correspond to the combination in quadrature of all the known uncertainty sources (ion intensity statistical uncertainties and those derived from the gas pressure and temperature determination, see Section 4 for details).

**Figure 8:**
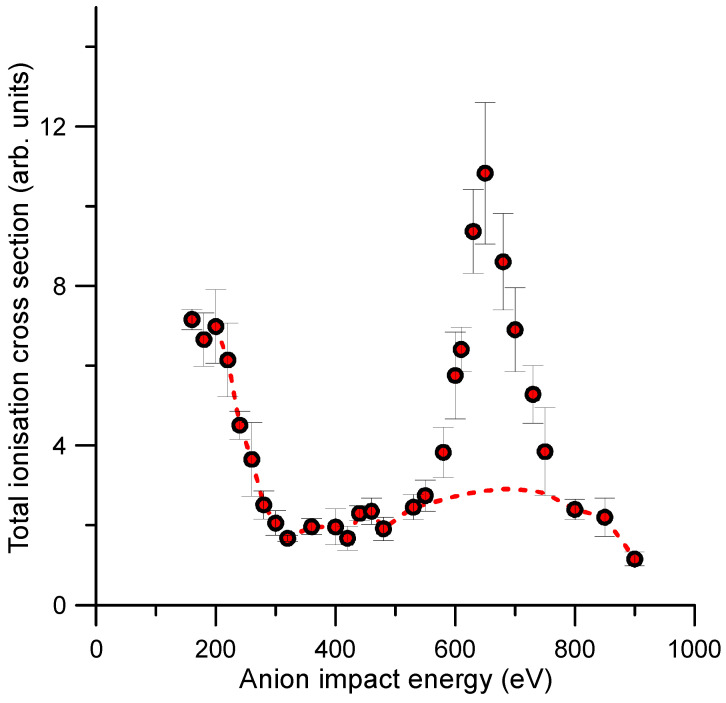
Relative total ionisation cross sections (TICS) accounting for all the cationic species generated by single O_2_^—^–C_6_H_6_ collisions for impact energies ranging from 160 up to 900 eV.

The total ionisation cross sections (TICS) derived by adding the intensities of all detected cationic species generated by the anion–molecule collisions considered in this study are shown in Figure 8. In such figure, the TICS present a prominent feature from 550 eV to 800 eV. Note that these species included the heavier adducts (*m*/*z* = 94–95, *m*/*z* = 110), whose formation process is described in Ref. [16]. According to the fragmentation pattern shown in Figure 7 and the above theoretical justification, we deduced that this prominent peak was mainly due to the fragmentation of these heavier adducts which mainly formed for impact energies from 550 to 800 eV. The dashed red line in Figure 6 represents the contribution to the TICS of the fragments coming from the dissociation of the parent ion, i.e., the TICS values we would obtain if these adducts did not form.

## 3. Discussion

The energy dependence of the TEDCS showed a quite flat behaviour except for two relative maxima and a local minimum, cross section values which were not clearly defined within the quoted uncertainty limits. These could be due to specific electronic excitations of the target molecule, but no theoretical evidence has been found to support such assumption. However, the relative TICS energy dependence showed a prominent local maximum in the cross section values for impact energies between 550 and 800 eV. According to the present theoretically predicted ionization and further fragmentation patterns, this local maximum mainly formed by the fragmentation of the heavier adducts formed by the aggregation model we previously proposed in Ref. [16]: a sudden double ionization of benzene and the subsequent electrostatic attraction between the dication and the anion formed a stable covalently bonded C_6_H_6_O_2_^+^ molecule, which evolved towards the formation of benzene–diol conformers. Here, we demonstrated that the fragmentation of these heavier molecules led to the formation of those species that mainly contributed to the local maximum in the total ionisation cross section. Thus, these results strongly support our previous adduct formation model, verifying the validity of the mechanism proposed in Ref. [16].

## 4. Materials and Methods

### 4.1. Experimental Setup and Procedure

The experimental setup used in this study was already described in detail in refs. [16,17]. Here, just a brief description with some improvements and modifications will be given. The experimental apparatus consisted of a sequence of high-vacuum chambers (8.4 × 10^−7^ mbar background pressure) connected to each other: a projectile chamber, a collision chamber and a transmission chamber. A schematic diagram of the apparatus is shown in Figure 1 of Ref [16].

The oxygen anionic beam was produced inside the projectile chamber through a plasma discharge process. The plasma pulse was generated within a hollow cathode discharge mechanism by applying −500 V to the cathode while the anode was grounded. The negative ions formed in the afterglow region, once the plasma-generated species de-excited, and secondary electron attachment and/or charge exchange processes occurred. A commercial Parker pulsed valve (VAC1250) was used to introduce the projectile (O_2_) into the vacuum chamber. It operated at 350 μs pulse width in an 80 ms duty cycle and at a gas pressure of typically 500 mbar. The background pressure in the projectile chamber during the pulsed valve operation was maintained below 10^−5^ mbar. Afterwards, the projectile beam left the discharge chamber, being focused and deflected towards the collision chamber through a combination of an Einzel lens (L1), placed just after the anode, and XY deflecting plates (D1 and D2), placed at the entrance of the second chamber (see Figure 1 of Ref. [13]). In the same region of the deflection plates, two magnets were placed outside the chamber in order to avoid stray electrons passing to the collision region. The oxygen anionic beam projectile reached the collision chamber entering through a 2 mm diameter hole after being focused by an Einzel lens (L2) and travelling into the gas cell (GC). The GC was a 36 mm-diameter and 27 mm-length cylindrical chamber, where a negative or positive voltage was applied to accelerate or decelerate the anion beam to define the kinetic energy of the O_2_^−^ anions and, therefore, the collision energy. During all measurements, a negative variable voltage of 200–280 V was applied to the central electrode of L2 to increase the beam’s intensity in the collision region.

The molecular interactions occurred in the GC when the molecular target was introduced at low pressure (<2 mTorr) through a 15 mm diameter aperture controlled by a sapphire leak valve. The positive ions formed in the GC were extracted and accelerated by means of an extractive plate system, through a perpendicular 1.40 m-long time-of-flight (TOF) mass spectrometer, placed over the collision region, where ions were mass-analysed and detected by a microchannel plate (MCP1) operating in single-pulse counting mode. The extractive parallel plates (E1, E2) were connected to the positive (0 to +900) and negative (0 to −900) V1 and V2 pulsed voltages, respectively. Pulse voltages and durations were optimized to ensure a total and uniform ion extraction but maintaining a reasonable mass resolution. The mass resolution used in this study was limited by the length of the hollow cathode. The uncertainty in the position where anions formed along the cathode was converted into a time of flight, and therefore mass, an uncertainty which determined our inherent mass resolution limit (Δ*m*/*m* = 0.05).

Under the GC, a homemade electron gun provided an energy-controlled electron beam (0–500 eV electron incident kinetic energy) entering the interaction region normal to the anion beam and opposite to the TOF mass analyser. The electron gun was not strictly necessary, but it was useful to analyse the molecular composition of the background and the gas target, as well as to heat the chamber to facilitate its evacuation when needed. It also provided a reference electron-induced fragmentation spectrum to compare with that produced by anion collisions.

A second microchannel plate (MCP2) was placed in the transmission chamber (see Figure 1 of Ref. [16]), 10 cm above the normal plane of the beam and 0.47 m from the hollow cathode source. The anionic beam was there repelled by a continuous −250 V voltage applied to E3 (E4 is grounded) towards the MCP2 detector which monitored the primary anion beam intensity (Figure 1). The MCP2 detector signal was also used to determine the primary beam energy distribution by using a set of three grids (G2) as a retarding field analyser placed at the entrance of the transmission chamber. A typical TOF mass spectrum of the projectile anionic beam is shown in Figure 2, comprising three features assigned to O^−^, O_2_^−^ and O_3_^−^, where O_3_^−^ intensity was about 50% of O_2_^−^ intensity. By tuning the extractive pulses, we could select the area of the primary beam which was really generating the positive ion fragments being mass-analysed (see refs. [16,17] for details).

In this study, we obtained the total electron detachment cross sections (*σ*_t_) for O_2_^−^ collisions with O_2_ by measuring the primary anion beam attenuations as a function of the target O_2_ pressure from 0 to 2 mTorr. The absolute pressure value was measured by two capacitance manometers (MKS Baratron 627B) placed at opposite sides of the GC (PGC1 and PGC2, respectively), normal to the primary beam direction, so ensuring that pressure gradients were not affecting the present measurements. Under these conditions, the total electron detachment cross-section value was derived from the Beer–Lambert attenuation law:

(1) $$I=I_{o}\exp\left(-\frac{Pl\sigma_{t}}{kT}\right)$$

where *I* is the transmitted anion intensity trough the GC, *P* is the O_2_ pressure [P_GC_ = (P_GC1_ + P_GC2_)/2], *I*_o_ is the initial intensity (no gas in the GC), *l* is the effective path length of the collision chamber (36 mm), *k* is the Boltzmann constant, and *T* is the absolute temperature. The O_2_^−^ transmitted intensity was measured with a single-ion counting system (see Ref. [16] for details) as a function of the O_2_ gas pressure. For each measurement, pressure varied from 0 to a maximum value (P_max_) which was chosen to ensure we were measuring the transmitted intensity under single-collision conditions [16]. According to the above expression (Equation (1)), for different impact energies, transmitted intensities, as a function of pressure, follow an exponential law with a slope directly providing the *σ*_t_ value.

Moreover, the total ionisation cross sections (*σ*_+_) can be derived from the ratio between the total positive ion detected intensity and the primary anion intensity, according to the expression:

(2) $$\sigma_{+}=\frac{I_{+}}{I_{-}}\frac{1}{nl}$$

where *I*_+_ is the total positive ion intensity as recorded by the MCP1 detector, I_−_ is the O_2_^−^ intensity measured by the MCP2 detector (transmitted O_2_^−^ intensity), *n* is the molecular gas density derived from the gas pressure (*P*_GC_) and the temperature (*T*) by assuming an ideal gas behaviour, and *l* is the effective ion extraction length which is supposed to be the diameter of the ion extraction aperture (2 mm). Under these conditions, Equation (2) provides absolute values of the cross sections. In the conditions of this experiment, the counting efficiency of both MCP1 and MCP2 was about 100%. However, as we could not ensure that the primary beam intensity detected by MCP2 corresponded to the actual intensity of this beam in the gas cell, only relative values of the ionization cross sections, as a function of the anion impact energy, could be obtained.

### 4.2. Uncertainty Analysis

Concerning the total electron detachment cross-section, statistical uncertainties were constrained below 5% by repeating at least 5 times the anion attenuation measurements and their corresponding data acquisition and analysis procedures for each impact energy considered in this study. The accuracy of the pressure determination was given by the uncertainty limit of the MKS-Baratron gauge (1%, according to the manufacturer’s data). However, due to pressure gradients in the gas cell, we considered the gas pressure as the average of those measured at both sides of the cell, so introducing an additional error contribution to the experimental cross sections of about 8%. By adding in quadrature all the known error sources and statistical uncertainties, a total uncertainty limit within 6–10% was determined for the absolute electron detachment cross sections. With respect to the relative ionisation cross section, instabilities in the hollow cathode discharge led to statistical uncertainties within 8 and 20%, depending on the impact energy.

### 4.3. Computational Details

Calculations were performed in the framework of the Density Functional Theory (DFT). The Gaussian16 package [18] was used to calculate stationary points of the potential energy surface and to perform molecular dynamics (MD) simulations. In order to carry out the MD, we used the Atom-Centered Density Matrix Propagation (ADMP) method [19,20,21]. In this method, nuclei move classically in the potential computed at a quantum level, in our case using the DFT. We chose the M06-2X functional [22] because it is adequate for systems with main group elements, including weak interaction forces, in combination with the 6-31++G(d,p) basis set [23,24,25], which includes diffuse functions, thus allowing a proper description of the electron density far from the nuclei. We considered singly ionized complexes with doublet spin multiplicity. Trajectories with the ADMP method were propagated up to 200 fs, with a time step of 0.1 fs and using a fictitious mass of µ = 0.1 amu, thus ensuring the adiabaticity in the dynamics. We ran trajectories with four different values of internal excitation energy, E_exc_ = 5, 15, 25 and 40 eV. In each trajectory, E_exc_ was randomly redistributed among the nuclear degrees of freedom. This method has been used with success in the past to study the fragmentation dynamics of ionized molecules and clusters of different nature [26,27,28,29,30,31,32,33].

## 5. Conclusions

Novel experimental total electron detachment cross sections for O_2_^−^ collisions with benzene molecules were reported for impact energies ranging from 10 to 1000 eV as measured with a transmission beam technique. By analysing the positively charged species produced during the collisions, relative total ionisation cross sections were derived in the incident energy range of 160–900 eV. Relative partial ionisation cross sections for fragments with masses *m* ≤ 78 were also given in this energy range. From the mass analysis of the fragments, we confirmed that the heavier compounds (*m* > 78) discussed in Ref. [13] formed for impact energies between 550 and 800 eV. By performing molecular dynamics calculations within the framework of the Density Functional Theory (DFT), we demonstrated that the fragmentation of these heavier compounds reinforced the creation of *m*/*z* = 39–42, 50, 60 u cations, which contributed to the formation of a local maximum in the total ionisation cross section within this energy range (550–800 eV). We did not find any previously published data to compare with the present results.

## Figures and Tables

**Table 1:** Experimental total electron detachment cross sections in SI units (10^−20^ m^2^). Total uncertainty limits are also included.

Energy (eV)	Total Electron Detachment Cross Section (10^−20^ m^2^)	Uncertainty Limit (%)
10	33.93	7.8
20	34.46	6.7
40	36.20	6.0
60	34.46	6.2
80	34.51	6.7
100	35.02	8.4
120	33.91	7.6
140	34.11	9.7
160	32.51	6.7
180	32.40	5.2
200	32.39	7.1
220	31.89	8.9
240	32.12	6.6
260	31.92	9.6
280	30.65	7.9
300	30.22	7.9
320	29.42	6.6
340	30.03	5.6
360	30.27	6.8
380	31.72	8.1
400	33.60	6.7
420	32.10	5.5
440	32.43	7.9
460	32.47	5.6
480	33.93	8.0
500	34.84	6.1
600	34.97	6.1
700	35.40	6.0
750	35.65	7.3
800	35.49	6.2
900	35.85	7.6
950	35.75	5.8
1000	36.78	5.9

## Data Availability

Not applicable.
